# Contrecoup epidural hematoma: a rare case report

**DOI:** 10.11604/pamj.2022.41.169.31986

**Published:** 2022-03-01

**Authors:** Farhad Bal’afif, Donny Wisnu Wardhana, Tommy Nazwar Alfandy, Ariel Jesse

**Affiliations:** 1 Division of Neurosurgery, Department of Surgery, Brawijaya University, Saiful Anwar Hospital, Malang, Indonesia,; 2 Department of Surgery, Brawijaya University, Malang, Indonesia

**Keywords:** Contrecoup, epidural hematoma, dura, case report

## Abstract

Epidural hematoma (EDH) is defined as a traumatic accretion of blood separating the dural membrane and the internal table of the skull that caused from contact bending or skull fracture. The cases of contrecoup EDH are as not common, and there are just 10 recorded cases on this. As a result of its uncommonness, we disclose one subject of a 33-year-old man having countrecoup EDH who suffered from a head trauma caused by falling from 4 meters high. The main symptoms were decreasing consciousness and vomiting. Clinical findings showed a Glasgow Coma Scale score of 9, laceration in the left parietal region with stable hemodynamic. Head Computed tomography showed a large EDH in the right frontal and temporal region with coronal suture diastasis. The patient immediately underwent surgery, and craniotomy with evacuation of extradural hematoma were performed. This case presents that a force, which creates an angle, can propagates and causes opposite coronal suture diastasis and makes contrecoup EDH.

## Introduction

EDH generally result from a coup head injury. However, the cases of contrecoup EDH are uncommon, also until recently, no more than 10 occurrences are disclosed [1]. Interestingly, patients with EDH contrecoup are predominantly females, relatively at old ages, high delay in appearance, and a high frequency of frontal involvement. At the age of 10-19 is when the occurrence of EDH is noted to be at its highest pinnacle. EDH is commonly found in the temporoparietal and temporal regions, whereas contrecoup EDH is often located in the frontal region (70%). It is because the dura mater of the lateral frontal region is not difficultly separated from the inner table, similar to what is found amid procedures of craniotomy. Repeated CT imaging is required, especially between 4-10 hours after the injury [1,2].

The mechanism factor of the EDH contrecoup is still imprecise. Dural separation and dural vascular injury because of cranial deformity due to impact forces are possible mechanisms contributing to the development of EDH. Since the early finding of contrecoup injuries is able to reduce complications of head trauma [2,3]. We present a 33-year-old man who lost consciousness and suffered from a head injury due to falling from high, who was suspected to have a contrecoup through CT scan but the final result consistent with fracture causes laceration of meningeal middle artery and EDH.

## Patient and observation

**Patient information:** a 33-year-old man was brought at 8.00 PM on July 14, 2021, to the Emergency Room of Saiful Anwar General Hospital Malang with a loss of consciousness after falling from 4 meters high when working as an electrician. The patient has a history of vomiting twice, but without convulsion, discharging blood from the ears, nose, or throat. Based on the history taking, there were no complaints about anosmia, ageusia, fever, cough, shortness of breath, or contact with COVID-19 patients.

**Figure 1:**
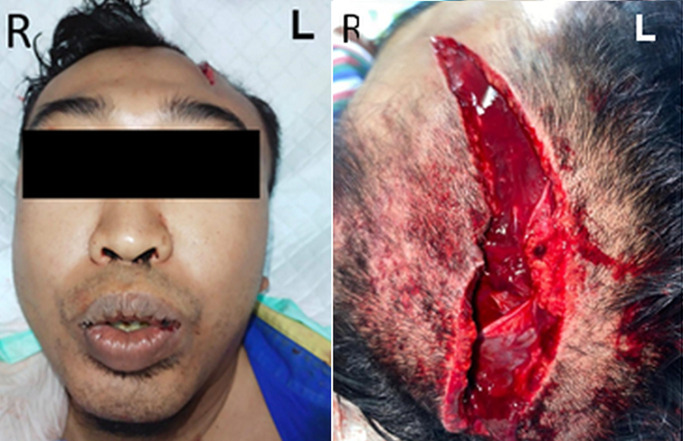
an irregular border, muscle-based, sized 10x3 cm lacerated wound on the left parietal region

**Clinical findings:** on clinical examination, there was a laceration 10 cm above the left parietal area (Figure 1). The laceration had an irregular border, muscle-based, dirty with no prolapsed brain and no cerebrospinal fluid leakage. His Glasgow Coma Scale (GCS) score was 9. The pupils were bilaterally isochoric and reacted. The examinations also revealed 110/60 mmHg blood pressure, 104 times/minute pulse, 20 times/minute breathing, 36,7OC temperature, and oxygen saturation 99% on room air.

**Timeline of the current episode:** the patient had a loss of consciousness after fell from high. His family brought him to the emergency unit in Saiful Anwar Hospital four hours after the accident by his family. The patient got a careful examination in the Saiful Anwar Hospital. The patient underwent a head computed tomography (CT) scan one hour since he came to the emergency room and got surgery 12 hours since admission. After successful surgery, the patient was hospitalized for eight days and then discharged.

**Figure 2:**
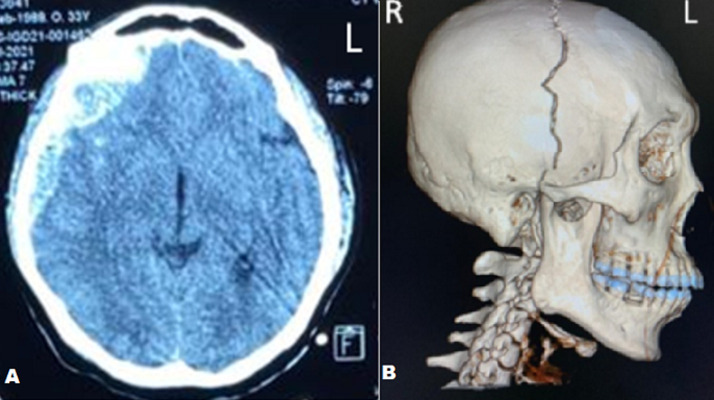
CT scan showing biconvex and crescent in right frontal and temporal region (A); 3D skull radiographic showing coronal suture diastasis widening up to the right temporal bone (B)

**Figure 3:**
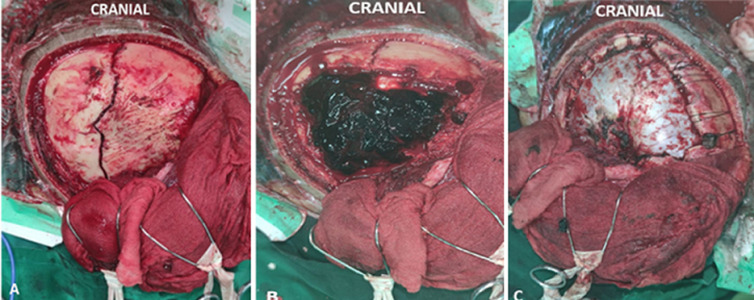
coronal suture diastasis (A); epidural hematoma (B); post evacuation hematoma (C)

**Figure 4:**
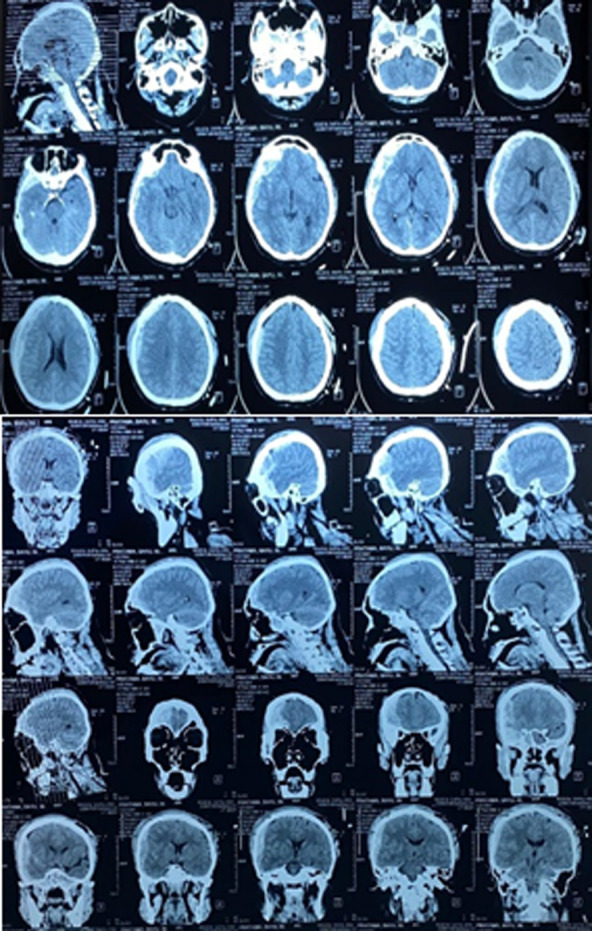
head CT before surgery showing Right frontal and temporal epidural hematoma and coronal suture diastasis

**Diagnostic assessment:** after the clinical examinations, the patient underwent a head computed tomography (CT) scan one hour since he came to the emergency room. CT scan showed a large epidural hematoma in the right frontal and temporal area (Figure 2) with coronal suture diastasis (Figure 3), cerebral edema, and a 5 mm midline shift to the left (Figure 4).

**Diagnosis:** from history taking and examination, the patient was diagnosed with moderate head injury with a GCS score of 9, open wound in the left frontoparietal area, frontotemporal cephalhematoma, 36cc of EDH in the frontotemporal area, SDH in the right temporobasal area with 10 slices of a 20 mm thickness, cerebral edema, coronal suture diastasis, and right temporoparietal area linear fracture.

**Therapeutic interventions:** the patient was managed with head elevation, oxygen supplementation, fluid administration, analgetics, tranexamic acid, mannitol 20%, anticonvulsant, and human antitetanus immunoglobulin. After informing his family that was checked, he was prepared for a surgery scheduled on July 15, 2021, in the morning. In the surgery, right frontal and temporal craniotomy with evacuation of the extradural hematoma was carried out. Coronal suture diastasis existed intraoperatively in the right frontal and temporal area (Figure 3).

**Figure 5:**
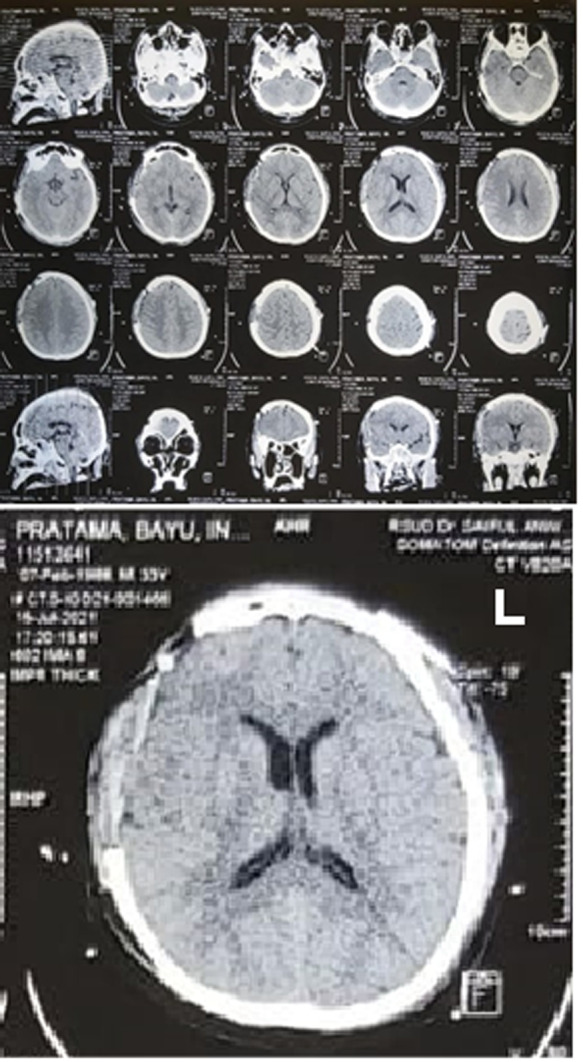
head CT after surgery showing improvement where the hematoma has been evacuated

**Follow-up and outcome of interventions:** a postoperative CT scan was executed and presented postoperative alterations (Figure 5). Mannitol and tranexamic acid were given in the postoperative stage. His GCS increased to 15 on the fifth day postoperative. The subject was monitored for vital signs, including general condition, heart rate, respiratory rate, body temperature, signs of increasing Intracranial pressure (ICP), GCS, lateralization, and response to therapy. The patient was hospitalized for eight days.

**Patient perspective:** as long as the patient is treated, the patient was delighted with the care and good service he received. He was optimistic that his condition will recover as before Informed Consent: the patient´s family was enlightened regarding the report of the reasons behind the peculiarity of his case. He provided briefed approval to let the authors apply his case for this case report.

## Discussion

EDH is responsible for 1% to 3% of all head injury incidences. It progresses just under the point of impact and is followed by a linear fracture in most occurrences; however, cases without fracture account for 10% to 20% [4]. The fatality rate ranges from 10% to 40%; in addition, it is an index of vigilance and adeptness of health care and hospital setups. Blunt head trauma accounts for the highest rate of a common factor of EDH, which ranges between 1% and 6%. Global fatality after a surgically medicated EDH is around 10%. Even though contrecoup contusion, as well as acute subdural hematoma because of escalation injury to the head, have been disclosed, no more than 10 contrecoup epidural hematoma occurrences are publicized in the article [3]. The most common cause of intracranial EDH is trauma. Despite the fact that infrequently spontaneous haemorrhage happens commonly, this occurs because of adjoining infections, dural vascular malformations, tumors, as well as blood coagulation afflictions. EDH is commonly coup lesions from discharging blood from a calvaria fissure or damage to the dural arteries. EDH may also have a venous origin, if not from diploic veins, it is from connecting fracture on their course into venous sinuses. These lower-pressure hematomas progress more moderately; in addition, are generally self-limited, however, no credible imaging indications can be applied to aid in their estimation, and this subtype ought to be categorized as malignant [4,5]. EDH adopts a lens-shaped of biconvex morphology since the periosteal layer of dura is peeled from the inner table of the skull by blood discharging [1,6].

The mechanism factor of the EDH contrecoup is still imprecise. Dural separation and dural vascular damage because of skull deformity due to impact forces are feasible factors contributing to the advancement of EDH. As stated by Jamieson, epidural contrecoup hematomas do not develop however are able to bilaterally occur if the midline sagittal sinus vessels are associated, or several strokes are endured [7]. Nevertheless, preceding this in 1991, an uncommon kind of bilateral epidural hematoma, one from straight injury and one other from a contrecoup impact, was stated by Balasubraminiam and Ramesh [8]. They noticed that local contortion at the site directly on the other side of the impact site contributed increase to the small pocket caused by the peeling of the dura. This contortion as well as the dependence impact formed by the removal of the former hematoma caused a contrecoup hematoma [2,3]. Miyazaki *et al*. also noted cases of bilateral coup and contrecoup epidural hematomas [9]. They deduced that the deformity of the skull caused by the pressure of the jolt caused the dural separation to result in a hematoma. This study presented a tiny air pocket beside the EDH, however intraoperatively as well as on imaging, no cranial fissure was spotted in the frontal and base regions. This discovery is additionally backed with an instance of *pneumocephalus* with no skull base rupture [10]. There are two suggested mechanisms to describe *pneumocephalus* with no craniofacial skull fissure, the two implicating low intracranial pressure resulting in the suction of air via the dural defect. The former mechanism includes vertical strain that generates a pressure slope in the cerebrospinal fluid (CSF) system, while the latter includes a ball valve impact that lets air get inside the skull base from several foramina or cervical cranial connection pathways [2,3].

The “classic” lucid interval including a quick loss of consciousness (LOC) was induced by the initial hit, accompanied by an awake stage as well as delayed disintegration because of the ICP and mass effect. It occurs in only a small number of instances, roughly 15% to 30%. From 20% to 55% of the patients are comatose when being admitted or shortly prior to surgery. Neurological discoveries rely on the precise area of the contrecoup injury. The majority of frontal lobe lesions will not appear in particular discoveries. However, temporal lobe trauma can generate speech changes; on the other word hemiparesis, and changes in mental status are often seen. Therefore, the patients may experience disorientation and confusion, and some patients present with seizures [1].

In this case, we proposed that the blunt object caused direct trauma to the head (left side) that made scalp avulsion and fracture of the skull; we assumed that the object contacted the skull with some angle. Therefore, it made an impact; the force propagated and caused diastasis to the coronal suture at the contralateral side (right side) until temporal bone at the right side. The fracture caused laceration of the middle meningeal artery at the right side, and right EDH occurred.

## Conclusion

EDH generally results from a coup head injury and contrecoup EDH is a rare entity. This case report demonstrates a blunt object that caused direct trauma to the head (left side), and resulted in scalp avulsion and fracture of the skull. We assume that the object contacts the skull with some angle, thus the force propagates and causes diastasis to the coronal suture at the contralateral side (right side) until temporal bone at the right side. The fracture causes laceration of the meningeal middle artery and EDH on the right side.
